# Electron tomographic analysis reveals ultrastructural features of mitochondrial cristae architecture which reflect energetic state and aging

**DOI:** 10.1038/srep45474

**Published:** 2017-03-30

**Authors:** Yi-fan Jiang, Shao-syuan Lin, Jing-min Chen, Han-zen Tsai, Tao-shih Hsieh, Chi-yu Fu

**Affiliations:** 1 Institute of Cellular and Organismic Biology, Academia Sinica, Taipei, Taiwan; 2 Graduate Institute of Molecular and Comparative Pathobiology, School of Veterinary Medicine, National Taiwan University, Taipei, Taiwan

## Abstract

Within mitochondria, the ability to produce energy relies upon the architectural hallmarks of double membranes and cristae invaginations. Herein, we describe novel features of mitochondrial cristae structure, which correspond to the energetic state of the organelle. In concordance with high-energy demand, mitochondria of *Drosophila* indirect flight muscle exhibited extensive intra-mitochondrial membrane switches between densely packed lamellar cristae that resulted in a spiral-like cristae network and allowed for bidirectional matrix confluency. This highly interconnected architecture is expected to allow rapid equilibration of membrane potential and biomolecules across integrated regions. In addition, mutant flies with mtDNA replication defect and an accelerated aging phenotype accumulated mitochondria that contained subsections of swirling membrane alongside normal cristae. The swirling membrane had impaired energy production capacity as measured by protein composition and function. Furthermore, mitochondrial fusion and fission dynamics were affected in the prematurely aged flies. Interestingly, the normal cristae that remained in the mitochondria with swirling membranes maintained acceptable function that camouflaged them from quality control elimination. Overall, structural features of mitochondrial cristae were described in three-dimension from serial section electron tomographic analysis which reflect energetic state and mtDNA-mediated aging.

Mitochondria are organelles that produce ATP, metabolites and lipids, as well as functioning to maintain Ca^2+^ homeostasis and mediate apoptosis^1^,^2^,^3^. These functions are delicately connected to the mitochondrial ultrastructure through the spatial arrangement of key molecules^2^,^4^,^5^. The outer and inner mitochondrial membranes enclose and define the inter-membrane space and matrix compartments. Invagination of the inner membrane forms cristae, where respiratory chain complexes are situated. These complexes pump protons from the matrix into the inter-membrane space by sequential redox reactions, thereby generating membrane potential across the inner membrane^2^. The proton gradient is then utilized to drive the phosphorylation of ADP to ATP through the rotary turbine-like ATP synthase (complex V)^2^. Thus, cristae morphology provides the infrastructural basis for proton exchange and is tightly linked to the energy production capacity of the mitochondria.

To carry out various cellular processes, mitochondria house over 1500 proteins which are encoded by nuclear DNA, synthesized in the cytoplasm and translocated to specific compartments of the organelle^6^. Mitochondrial DNA (mtDNA) encodes only thirteen gene products, all of which are incorporated into respiratory complexes and utilized in oxidative phosphorylation. Accumulation of mutations and deletions in mtDNA, which exists as multiple copies in a single mitochondrion, may contribute to physiological aging and a wide range of disorders^7^,^8^. During replication and transcription, DNA topoisomerase relieves topological strains introduced to the DNA double helix^9^. In *Drosophila*, depletion of mitochondrial-targeted topoisomerase IIIα, through truncation of the N-terminal mitochondria targeting signal, results in mitochondrial dysfunction, accelerated aging, and infertility^9^. At five weeks of age, the mutant flies (R_M1L_) were shown to accumulate approximately threefold more mtDNA deletions and an increased amount of replication related concatamers compared to the Rescue wild-type (R_WT_) flies^10^. Because R_M1L_ *Drosophila*, mimic mtDNA stress-accelerated aging, the model offers a great opportunity to investigate how the accumulation of mtDNA defects over time impacts the structure and function of an individual mitochondrion as well as the mitochondrial network.

In this study, we used three-dimensional (3D) reconstructions from electron tomographic data to examine mitochondrial ultrastructure in the indirect flight muscle (IFM) of wild-type (WT), R_WT_ and R_M1L_ flies during aging.

## Results

### Mitochondria of Drosophila IFM form an integrated connective cristae network in three dimensions

**Figure 1:**
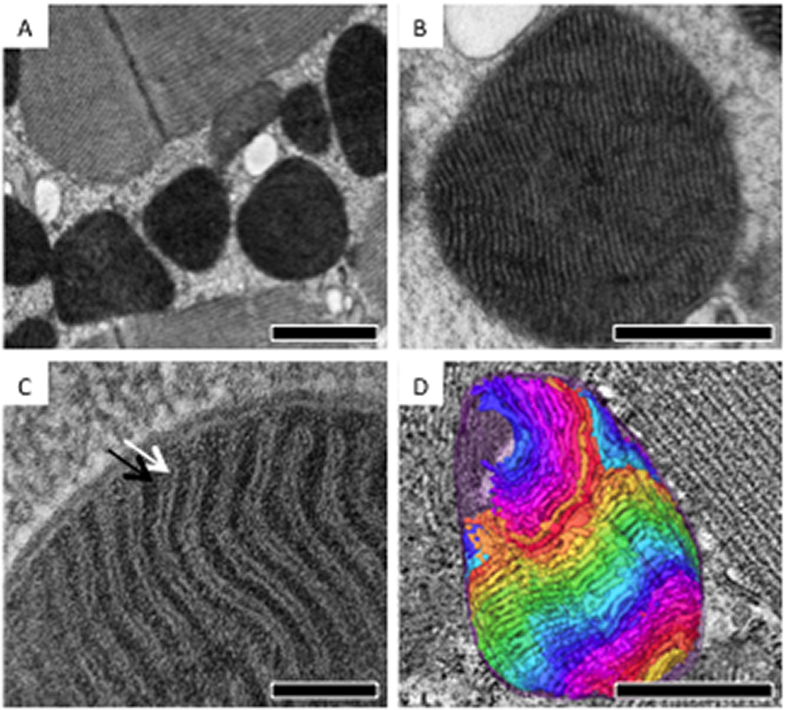
*Drosophila* IFM mitochondria containing densely packed lamellar cristae. (**A**–**C**) Representative thin-section EM images of *Drosophila* IFM showing mitochondria with double membranes and densely packed lamellar cristae. (White arrow, cristae; black arrow, matrix) (**D**) 3D ultrastructural segmentation of a mitochondrion that was reconstructed by serial-section electron tomography. Volume segmentation of cristae was displayed in arbitrary color rendering. Scale bar: 1 μm (**A**), 0.5 μm (**B**), 0.1 μm (**C**), and 0.5 μm (**D**).

*Drosophila* IFM was dissected and subjected to high-pressure freezing and freeze substitution (HFP/FS) in order to better preserve the tissue ultrastructure^11^. Thin-section EM images showed that IFM mitochondria distribute along, and align roughly in parallel with muscle fibers, creating the appearance of alternating layers of mitochondria and muscle fibers in longitudinal sections (Fig. 1A). IFM mitochondria contain outer, inner boundary membranes and cristae invaginations (Fig. 1B,C). Extremely compact lamellar cristae were developed in concordance with the high-energy demand of IFM. Although thin-section TEM provided informative morphological insights, two-dimensional images can only show the ultrastructure of a particular cross-section. Therefore, the ultrastructural organization in 3D remained obscured.

In order to visualize the architecture in 3D, we applied electron tomography, in which a series of tilt images of the section were collected and back-projected to construct 3D tomograms^12^. To obtain reconstructions covering the entire mitochondrial volume (~2.5 μm-thick), tomograms were collected from serial sections of the specimen, after which they were reconstructed and combined (Fig S1). Joint serial tomograms that span the volume of entire mitochondria were subjected to volume segmentation and structural analysis (Fig. 1D).

**Figure 2:**
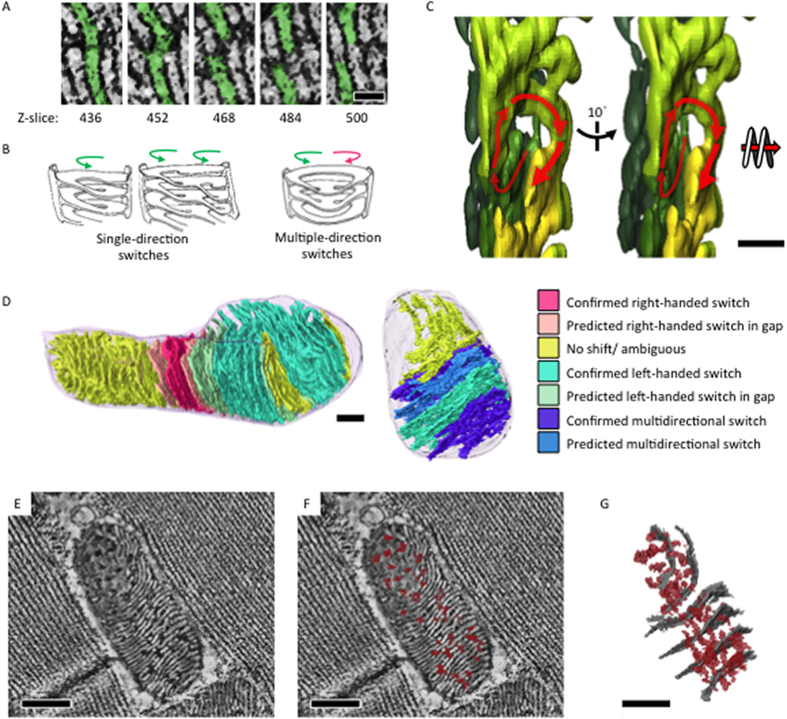
Integrated intra-mitochondria cristae and matrix network in 3D. (**A**) Slices of mitochondrial electron tomographic reconstruction showing switches between lamellar membranes through the z-axis. (**B**) Carton illustrations of the observed cristae switching patterns of right handed and/or left-handed spirals. (**C**) Tomographic segmentation illustrating a left-handed spiral in 3D (**D**) Jointed tomograms were subjected to volume segmentation. Cristae switching patterns were analyzed and color-rendered on the segmentation model accordingly (**E**,**F**). Tomographic slice with lateral matrix confluency (dark densities, marked red) across cristae membranes (white densities). (**G**) Segmentation model of the tomogram in (**E**) showing lateral matrix confluency (in red) and representative cristae (in gray). Scale bar: 50 nm (**A**), 200 nm (**C**), 300 nm (**D**), and 300 nm (**E**).

Mitochondria of *Drosophila* IFM have extremely compact lamellar cristae. Cristae membranes are approximately 17 nm in thickness and confine the matrix to approximately 13 nm in width. In 3D, cristae membranes are not separated as isolated invaginations that only connect at the inner boundary membrane; rather they form a reticulated structure with extensive inter-cristae connections and integrated webs (Movie S1). Figure 2A illustrates a typical connection between lamella. In the leftmost panel, a single crista has been subdivided into two pieces. Progressing through the z-stack to panels on the right, both the lower and upper portions switched connections and adjoined to neighboring crista. Hereafter, we refer to this type of 3D structure as a switch, and observed that the switches between lamella will create directional spirals (Fig. 2A–C). Even though the reconstructed volume covered an entire mitochondrion, physical loss of material between sections and the missing-wedge effects from tomographic data collection still left gaps and ambiguities in some regions of the joint tomograms^12^. However, analyzing data with sufficient resolution showed that cristae from an individual mitochondrion could interconnect through combinations of single- or multi-directional switches (Fig. 2A–C). Moreover, subsections of the mitochondria could be defined as predominantly containing right-handed or left-handed spiral-like cristae connections, or sophisticated inter-cristae webs that involve multiple switches of single crista or switches that bypass the adjacent crista and complete with the second nearest one (Fig. 2D). Of note, the switching pattern in the gap caused by the material loss during tissue sectioning or missing wedge artifact during data collection was predicted based on the switching pattern of the tomograms sandwiched the gap. The lamellar stacks of cristae established an integrative network through inter-cristae membrane switches, in addition to the connection via the inner boundary membrane. Cristae breaking and switching points indicate points of confluence through which matrix components may be distributed across the width of multiple cristae instead of being confined to single layers (Fig. 2E–G, Movie S2). We refer to these extensions between lamellae as confluence basins. As a result, the matrix was able to continuously extend through uninterrupted channels both laterally and longitudinally relative to the reference of the outer and inner boundary membranes. We refer to this state of complete lateral and longitudinal interconnection as bidirectional matrix confluency.

Spiral-like cristae switches and bidirectional matrix confluency create robust, physical, intra-organelle connectivity that is expected to facilitate swift equilibration of membrane potential and biomolecules throughout the mitochondrion in response to changing demands or inter-mitochondrial dynamic changes. In addition, cristae switches are composed with curved membrane ridges, which in contrast to flat cristae surfaces, have been shown to preferentially accumulate ATP synthase in many species^13^,^14^. Therefore, it is likely that the cristae switches of IFM mitochondria provide sites for ATP synthase integration in the cristae and thus facilitate ATP production.

### Mitochondria with onion-like swirling membrane accumulated in aged M1L Drosophila

**Figure 3:**
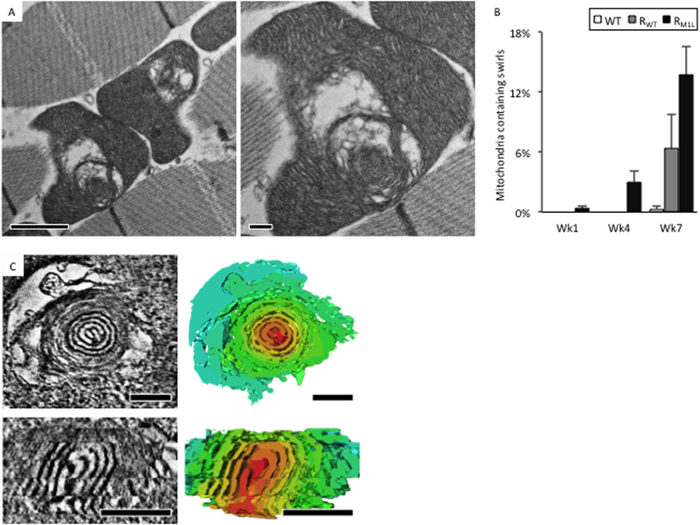
Mitochondria with onion-like swirling cores accumulated in R_M1L_ flies during aging. (**A**) Representative thin-section EM images of mitochondria with swirling membranes and surrounded swollen spaces. (**B**) The population of swirling mitochondria in WT, R_WT_, R_M1L_ at week 1, 4, and 7. (**C**) Representative tomographic slices and the corresponding segmentation (right panels) showing a cross-sectional view (top) and a longitudinal-section view (bottom) of a swirling core. Volume segmentation was displayed in arbitrary color rendering to highlight the swirling core. Scale bar: 200 nm. Scale bar: 1 μm (**A**, top panel), 0.2 μm (**A**, bottom panel), 200 nm (**C**).

In order to examine mitochondrial cristae morphologies that may be associated with the accelerated aging phenotype of mtDNA replication deficient flies, IFM of R_M1L_, R_WT_ and WT were dissected at weeks 1, 4 and 7 and used for thin-section TEM analysis. For each time point, 3–6 specimens were analyzed. Approximately 100–300 mitochondria of each specimen were classified by their morphology and calculated the percentage of appearance. The mean and standard deviation were computed with data of different specimens at each time point and plotted in Fig 3B. As *Drosophila* aged, mitochondria with swirling membrane morphology (Fig. 3A) were more frequently found in tissue from R_M1L_ compared to R_WT_ and WT (Fig. 3B). In addition, mitochondria with other atypical morphologies were observed more frequently as flies aged. These atypical morphologies included mitochondria with dispersed cristae, sparse matrix, and ruptures of the outer membrane, among others (data not shown).

To characterize the 3D architecture of swirling membranes within mitochondria, serial section electron tomography was performed. Tomograms were reconstructed, combined, and segmented (Fig. 3C, Movie S3). Swirling membranes were observed to contain onion-like core structures with condensed membranes compacted in layers (Fig. 3C). The compacted membranes remained connected between layers, which are highly reminiscent of the membrane switches and confluence basins between lamellar cristae. Onion-like membranes converged in the center of the swirl and extended to the exterior portion of the swirl where normal lamellar cristae were found. This phenomenon is depicted in the longitudinal section shown in Fig 3C. Often, enlarged swollen spaces surrounded the onion-like cores. In these swollen spaces, there was a marked loss of matrix components (Fig. 3C).

### Compromised molecular composition and functionality of swirling membranes

**Figure 4:**
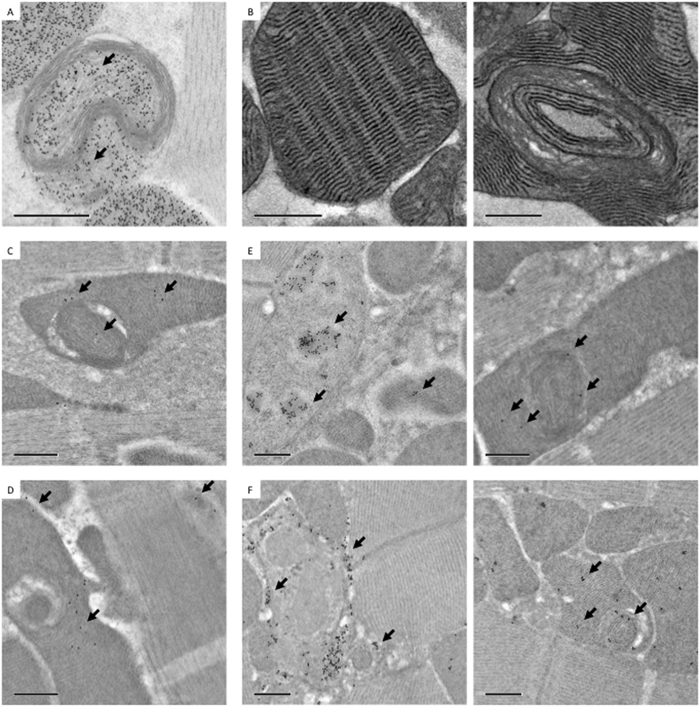
Mitochondria with swirling membranes retained partial function in sub-sections containing lamellar cristae. (**A**) Immuno-EM analysis of ATP synthase showing an absence in swirling membranes and presence in lamellar membranes (arrow: immune-gold). (**B**) COX activity assay showing weak staining in swirling membranes and strong staining in lamellar membranes. Immuno-EM analysis of PDHA1 (**C**) and SOD2 (**D**) showing preferential distribution in regions containing lamellar cristae compared to swirling membranes. Of note, the labeling was mostly absent from surrounding muscle tissue that showed relative good labeling specificity. Immuno-EM labeling of dsDNA (**E**) and DNA-RNA hybrids (**F**) showing the presence of both species around swirling membranes. Left panels of (**E** and **F**) showed regions of nuclei as a demonstration of labeling specificity. Scale bar: 0.5 μm.

To address whether the swirling membranes in mitochondria were still functional, immuno-EM was utilized to locate functional proteins in relation to the membrane ultrastructure. Figure 4A shows that ATP synthase, which is partially encoded by mtDNA and localizes to the cristae, was absent in condensed swirling membranes. However, ATP synthase was distributed in areas containing normal cristae architecture, as well as in regions that were enclosed by abnormal swirling membranes (Fig. 4A). The western-blot analysis of ATP synthase of R_WT_ and R_M1L_ at week 1, 4, 7 showed no dramatic alteration in the total protein expression level, as only portions of swirling membrane present (Fig. S2). Various studies have suggested that ATP synthase is essential to the formation and maintenance of cristae ultrastructure, in addition to its function in ATP synthesis^15^,^16^. The absence of ATP synthase on swirling membranes may reflect its critical role in establishing normal cristae architecture.

Cytochrome c oxidase (COX) is a respiratory complex which is also partially encoded by mtDNA. COX activity staining is performed by coupling the oxidation of diaminobenzidine (DAB) to cytochrome c oxidation. Oxidized DAB forms osmiophilic precipitants that can be visualized by EM. Typical lamellar cristae showed dark COX staining compared to swirling membranes that were stained weakly for COX activity (Fig. 4B). Surprisingly, the exterior of swirling membranes that contained normal lamellar cristae retained COX activity. This result indicates that mitochondria with swirling membranes are not completely dysfunctional, but instead, the normal cristae within the organelle retain function. The COX staining data were consistent with immuno-EM results showing that functional proteins encoded in nuclear DNA and translocated to mitochondria, such as pyruvate dehydrogenase (PDHA1) and superoxide dismutase (SOD2), also distributed to regions with normal lamellar cristae (Fig. 4C,D). The low labeling efficiency likely resulted from suboptimal quality of the antibodies as well as the loss of antigenicity during specimen preparation, even though we had applied HPF/FS to minimize the loss. Nevertheless, the libeling was mostly absent from the surrounding muscle tissue that indicated relatively good labeling specificity.

Since R_M1L_ flies have compromised mtDNA replication, the distribution of mtDNA in mitochondria with swirling membrane ultrastructure was inspected by immuno-EM. Both dsDNA and DNA/RNA hybrids were detected in and around swirling membranes (Fig. 4E,F). We speculate that localized alteration of cristae architecture and molecular composition might result from nearby defective copies of mtDNA, since R_M1L_ flies are known to accumulate deletions and unresolved replication concatamers over time^10^.

### Mitochondria with swirling membranes impact the mitochondrial reticulum

**Figure 5:**
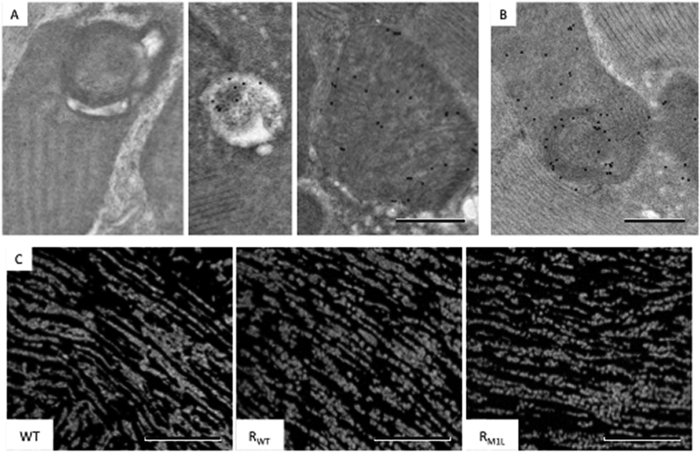
Mitochondrial reticulum in R_M1L_ flies. (**A**) Immuno-EM analysis of ubiquitin showing an absence in swirling mitochondria (left), and presence in autophagosomes (middle) and in mitochondria with sparse cristae (right). (**B**) Immuno-EM analysis of cytochrome c showing its presence in swirling mitochondria. (**C**) Immunofluorescent analysis of mitochondrial morphologies in WT, R_WT_ and R_M1L_ IFM at week 7. Scale bar: 0.5 μm (**A**,**B**), 20 μm (**C**).

Cells utilize multiple mechanisms to maintain a functional pool of mitochondria, including the regulation of autophagy or mitochondrial dynamics in response to changing physiology. One essential strategy to remove dysfunctional mitochondria is through mitophagy. Mitochondria with low membrane potential (an indicator of malfunction) are targeted to the autophagosome by PINK1 and Parkin dependent ubiquitinylation of constituent proteins^17^. To address whether mitochondria with swirling membranes were destined for mitophagy, the level of ubiquitination was characterized by immuno-EM. No mitochondria with swirling membranes were detected as ubiquitinated (Fig. 5A). However, some mitochondria with sparse cristae morphology or autophagosomes were tagged by ubiquitin (Fig. 5A). Of note, the frequency of ubiquitin immuno-labeling was lower than we expected. This may be the result of either a low affinity antibody or that ubiquitination represents a transient event that is difficult to capture. Nevertheless, the data suggested that cells recognized mitochondria with swirling membranes as being normal, so long as the area of functional lamellar cristae can maintain acceptable membrane potential of the organelle.

Mitochondrial homeostasis is maintained by fusion and fission (Westermann 2010, Hoppins 2014). Mitochondria of aged R_M1L_ IFM appeared to be more fragmented when assayed by immunofluorescent staining of thin-sections (Fig. 5C). The data imply that the quality control surveillance mechanisms recognize swirling mitochondria as suboptimal and therefore shift fusion/fission dynamics from fusion and toward fission.

Mitochondria regulate apoptosis by releasing mitochondrial cytochrome c to the cytoplasm and thereby activating the downstream caspase cascade^17^. Immuno-EM analysis showed that mitochondria with swirling membranes still contained cytochrome c at a comparable level with mitochondria of normal morphology (Fig. 5B). However, the data remained unconfirmed due to the lack of suitable antibodies against downstream apoptosis cascade.

### Mitochondrial cristae appear vacuolated and disintegrated in long-lived WT Drosophila

In long-lived WT flies (week 10 and 12), increased proportions of mitochondria showed cristae vacuolation and disintegration accompanied by the loss of matrix components compared to week 4 flies (Fig. S3B). Some mitochondria contained only reminiscent and fragmented cristae stacks. Immuno-EM analysis showed more sparse distribution of functional protein, such as ATP synthase, pyruvate dehydrogenase and superoxide dismutase 2, on the ultrastructure as the flies aged (Fig. S4A–C). Cytochrome c content was also slightly decreased, and ubiquitinated mitochondria and autophagosome structures were observed at a higher frequency. However total expression levels of functional proteins at week 10 were similar or slightly decreased compared to week 4 flies by western-blot analysis (Fig. S4D). Higher proportions of mitochondria appeared to be larger and fused together, suggesting an attempt to rescue function through fusion processes (Fig. S3A). Overall, the data demonstrate a pattern of global disintegration of architecture and lost function in super aged flies.

## Discussion

Mitochondria respond dynamically to changing energy demand and cellular physiology. Physical contacts have been reported to allow efficient communication with other organelles, such as with ER to regulate Ca^2+^ homeostasis, and facilitate transport of lipids and biomolecules^18^,^19^. In addition, mitochondria can organize as a reticulated super-structure that undergoes dynamic fusion and fission events to optimize collective cellular function^20^. In mouse cardiomyocytes, trans-mitochondrial coordination of cristae is achieved by forming inter-mitochondrial junctions. This process may facilitate the propagation of bioenergetic and apoptotic signaling throughout the mitochondrial reticulum^21^. In addition to these inter-organelle connections, in this paper, we describe intra-mitochondrial membrane switches between lamellar cristae that build spiral-like cristae webs and allow a physical means to achieve bidirectional matrix confluency. The extensive connections between cristae may also provide a structural foundation of cristae ridges for the incorporation of ATP synthase and also allow for rapid equilibration of membrane potential and biomolecules across integrated patches to increase the efficiency of energy production. However, we did not determine whether observed cristae breaks and switches resulted from intra-mitochondrial cristae remodeling that was dependent on inter-mitochondrial fusion. During inter-mitochondrial fusion, outer membrane fusion is followed by inner membrane fusion. Subsequent mixing of matrix proteins and inner membrane constituents then occurs to re-equilibrate the fused mitochondria^22^,^23^. It remains to be elucidated if intra-mitochondria cristae undergo dynamic remodeling independent of inter-mitochondria fusion/fission events.

**Figure 6:**
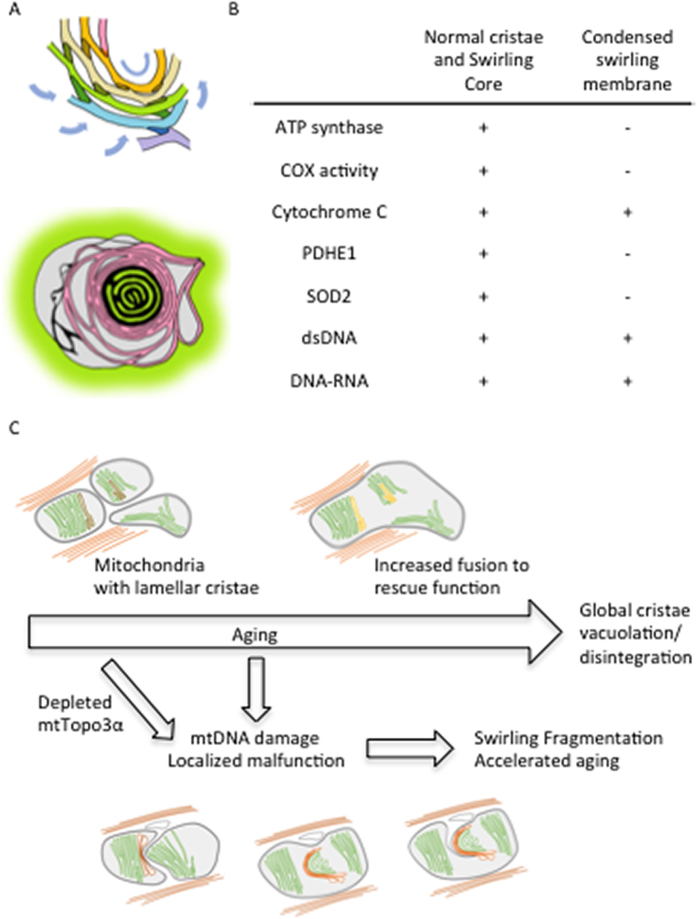
Hypothetical model illustrating the structural and functional consequences of mtDNA defects. (**A**) Proposed model illustrating a possible mechanism for the formation of swirling membrane. Localized loss of cristae proteins and function resulted in the collapse of membranes, loss of matrix components and swelling. The induced membrane curvature triggers a chain reaction of swirling of the cristae that were originally connected in a spiral-like manner. (**B**) A table summarizing the observed molecular composition of lamellar and swirling membranes. (**C**) Proposed model illustrating the mitochondrial ultrastructure changes during *Drosophila* aging. IFM mitochondria become larger during normal aging and eventually cristae become vacuolated and disintegrate. In R_M1L_ mutants lacking mitochondrial Topoisomerase IIIα, mtDNA defects in concordance with localized structural disruptions and malfunction lead to membrane swirling, mitochondrial fragmentation and accelerated aging.

mtDNA is required for mitochondrial function and therefore physical health and longevity, according to one widely-regarded aging hypothesis^7^,^24^. Over time, defective mtDNA copies and dysfunctional mitochondria accumulate, gradually becoming dominant in a cell and impairing cellular function^24^. In order to minimize the propagation of defective mtDNA during development, bottleneck selection on the basis of mitochondrial fitness, is employed to eliminate damaged or deleted mtDNA during oogenesis in *Drosophila*^25^,^26^. On the contrary, during adulthood, each mitochondria contains several copies of mtDNA as a mechanism of functional redundancy to minimize the impact of single mtDNA and maximize the function of the mitochondrial collective in a cell^7^. It has been proposed that damaged mtDNA and the surrounding impaired area undergo selective fission and are targeted for degradation by mitophagy^2^,^27^. However, in *Drosophila* R_M1L_ this quality control mechanism did not appear to be employed to eliminate impaired mtDNA and surrounding dysfunctional regions efficiently, even though R_M1L_ mitochondria were fragmented. Mitochondria with swirling membranes were previously reported in *Drosophila* under hypoxia or in mutants of compromised mitophagy, implying that they can accumulate as a result of inadequate quality control elimination^28^,^29^. The localized disruptions of ATP synthase distribution, cristae architecture and function are in concordance with the accumulation of defective mtDNA copies in R_M1L_. These disruptions could be expected to first produce local swelling of inter-membrane spaces, loss of matrix components and collapse of membranes, with further development to regional swirling morphology given the intrinsic curvature and connectivity of the lamellar membranes (Fig. 6A). Since mitochondria with swirling membranes still contained areas with normal morphology and function, the membrane potential of the organelles was probably maintained, and defective mtDNA copies were likely compensated for by functional copies and therefore hidden and tolerated in the mitochondrial network. The *Drosophila* R_M1L_ studies elucidated how defective mtDNA may be camouflaged and accumulate in the collective by producing self-contained regions of swirled membrane that minimize impact on mitochondrial structure and function. This process may be highly relevant during aging and follow as a result of the biological strategy of functional redundancy.

In summary, this study showed that normally aged WT flies displayed global cristae vacuolation and disintegration accompanied with increased fusion of mitochondria network. In contrast, augmented mtDNA damages in R_M1L_ flies caused localized malfunction and membrane swirling that led to mitochondrial fragmentation and accelerated aging process (Fig. 6C).

## Methods

### Fly strains and culture conditions

*Drosophila* strains Oregon-R-P2 WT, R_WT_, and R_M1L_ were cultured in an incubator at 25 °C and 60% humidity under 12 hr light and dark cycles. Female flies, which have larger IFM, were used for the studies. The culture density was controlled at 10 flies per tube. Fresh media and tubes were replaced every two days until sampling.

### Conventional specimen preparation for thin-section TEM

Fly thoraxes were isolated and immersed in a drop of fixative containing 2.5% glutaraldehyde in 0.1 M cacodylate buffer. IFM was dissected and subjected to a standard preparation protocol where specimens were fixed with glutaraldehyde and osmium, dehydrated with ascending concentrations of ethanol, infiltrated and embedded in Spurr’s resin, which was subsequently polymerized at 65 °C for 16 hr. Ultra-thin sections of 70 nm thickness were cut with an ultramicrotome (Leica EM UC7), mounted to 200-mesh copper grids, and stained with 2% uranyl acetate in 70% methanol for 10 min, followed by Reynold’s lead citrate for 4 min. The specimens were imaged with a FEI Tecnai G2 TF20 Super TWIN microscope operating at 120 kV. Six flies of three different strains were sampled at weeks 1, 4 and 7 to analyze mitochondrial ultrastructure.

### HPF/FS specimen preparation for electron tomography

IFMs were dissected in fixatives containing 2.5% glutaraldehyde in 0.1 M phosphate buffer, followed by a washing step of three drops of phosphate buffer and two drops of PBS with 20% BSA. The specimens were subsequently placed in a 3 mm A-type gold carrier with 0.1 mm indentation (16770152, Leica) filled with 20% BSA in PBS, and covered by the flat side of a B-type carrier (16770153, Leica). The carriers were loaded into a high-pressure freezer (Leica EM HPM100) according to manufacturer’s instructions. The carriers were subsequently released from the holder under liquid nitrogen and transferred to the chamber of a freeze-substitution device (Leica EM AFS2) pre-cooled to −140 °C and incubated for 96 hr at −90 °C in the FS cocktail (0.1% uranyl acetate, 2% glutaraldehyde and 2% osmium tetroxide in acetone).

Over the course of FS, the temperature of the chamber was raised at a slope of 5 °C/hr. The specimens were substituted with the FS cocktail at −60 °C for 12 hr, followed by at −25 °C for 12 hr, and washed with acetone at 0 °C three times for 1 hr each. The specimens were subsequently removed from the carriers using a needle, infiltrated and embedded in EMBed-812 resin at room temperature, which was polymerized at 65 °C for 16 hr.

### Serial-section electron tomography

Serial sections with thickness of 200 nm or 250 nm were prepared and collected on copper slot grids (2 × 0.5 mm oval slots) with carbon supports, on which overlaid with 10 nm fiducial gold pretreated with BSA. The grids were stained with Reynold’s lead citrate before the second layer of fiducial gold was applied. The specimens were imaged with FEI Tecnai TEM operating at 200 kV and the micrographs were recorded with a Gatan UltraScan 1000 CCD at 0.87 nm/pixel (9,600x). Tilt series from −60° to +60° with 2° increments were acquired using Xplore3D^TM^ (FEI). Double tilt series were collected using a double tilt holder (Model 2040 Dual-Axis Tomography Holder, Fischione). Serial tomograms were reconstructed, joined using IMOD, and segmented using Avizo 3D software (FEI).

### HPF/FS specimen preparation for immuno-EM labeling

IFMs were dissected in fixatives containing 4% paraformaldehyde, 0.25% glutaraldehyde in phosphate buffer and subjected to HPF/FS as described in the protocol for electron tomography with some modifications.

Immuno-EM specimens were freeze-substituted with 0.1% uranyl acetate in acetone at −90 °C for 58 hr (agitated every 8 hr), and warmed up to −45 °C at a slope of 5 °C/hr, washed with acetone three times for 1 hr each. The specimens were subsequently infiltrated through an ascending gradient of Lowicryl HM20 resin (10%, 20%, 40%, 60%, 80% and 90%, 8 hr for each concentration, and agitated every 2 hr). The chamber was further warmed up to −25 °C at 5 °C/hr. The solutions were replaced with 100% HM20 three times for 24 hr each (agitated every 2 hr). After adjusting the orientation within the carriers, ultraviolet polymerization was performed at −25 °C for 48 hr. The chamber was later warmed up to 20 °C (5 °C/hr) and exposed to ultraviolet radiation for another 48 hr.

After polymerization, the specimen blocks were detached from HPF carriers. 100 nm thick sections were prepared and placed on 200-mesh nickel grids for immuno-EM labeling.

### Immuno-EM labeling

Thin sections placed on nickel grids were blocked with 10% BSA in PBS for 20 min, and incubated with primary antibodies in incubation buffer (1% BSA in PBS) for 2 hr. Grids were subsequently washed with incubation buffer three times (10 min each). Secondary antibodies, goat anti-mouse IgG (EM.GMHL15, BB International) and protein A (EM.PAG15, BB International) conjugated to 15 nm gold particles, were used against the primary antibodies from mouse and rabbit respectively. Secondary antibodies at 20-fold dilution were applied and samples were incubated for 1 hr. After washing with PBS, the immune-complexes were fixed with 1% glutaraldehyde in PBS and washed three times with distilled water. The specimens were inspected by TEM operating at 120 kV (FEI Tecnai G2 TF20 Super TWIN).

The primary antibodies and applied dilution factors are listed as follows: mouse anti-dsDNA (500x, abcam ab27156), mouse anti-ATP5A (500x, abcam ab14748), mouse anti-Cytochrome C (8000x, abcam ab13575), mouse anti-PDHA1 (500x, abcam ab110334), mouse anti-Ubiquitin (1000x, abcam ab7254), mouse anti-DNA-RNA hybrid (500x, kerafast ENH001), and rabbit anti-SOD2 (500x, abcam ab13534).

### EM staining for COX activity

Fly IFMs were dissected and placed in drops of fixative containing 2% glutaraldehyde in PBS. The specimens were further fixed in fresh drops of fixative for 10 min and washed in three drops of PBS. The specimens were stained for 3 hr at 37 °C in a staining solution that contained 5 mg 3,3′-diaminobenzidine tetrahydrochloride, 9 ml sodium phosphate buffer (0.05 M, pH7.4), 750 mg sucrose, 20 μg catalase (dissolved in 0.05 M potassium phosphate buffer, pH 7.0), and 10 mg cytochrome c (dissolved in distilled water) at a volume of 10 ml. Subsequently, the specimens were washed with PBS for 1 hr and subjected to standard osmium fixation, dehydration, infiltration and embedded using Embed-812 resin. Thin-sections (70 nm) were cut and observed under TEM without further staining.

### Immunofluorescence Microscopy

The ultra-thin sections (100 nm) were cut from the blocks prepared for immuno-EM studies. The sections were air dried on the coverslips. Blocking and staining were performed as described in the immuno-EM method session. Mitochondria were labeled using antibody against ATP synthase (500x, abcam ab14748) and secondary antibody conjugated with fluorescent probe (500x, abcam ab150107). The coverslips with sections were then mounted with anti-fade mounting medium (ProLong Gold Antifade Mountant, ThermoFisher P36934) and observed under confocal microscope (Leica TCS SP5).

### Western-blot analysis

For each specimen, four thoraxes were dissected and homogenized by dounce tissue grinder in RIPA buffer containing protease inhibitor (cOmplete^TM^, Roche). Cellular debris were removed by centrifugation at 4 °C, 14000× g for 20 min. The supernatants were collected and the protein concentrations were determined by Pierce protein assay (Pierce 660 nm Protein Assay Reagent, ThermoScientific). 0.6 μg/well of proteins were loaded for SDS-PAGE and western-blot analysis.

The primary antibodies and the dilutions were as follows: mouse anti-ATP5A (50000x, abcam ab14748), mouse anti-Cytochrome C (10000x, abcam ab13575), mouse anti-PDHA1 (1000x, abcam ab110334), rabbit anti-SOD2 (10000x, abcam ab13534) and rabbit anti-alpha Tubulin (10000x, abcam ab18251). The secondary antibodies and the dilutions were as follows: anti-mouse IgG-HRP (2000x, Invitrogen 62–6520), or anti-rabbit IgG-HRP (5000x, abcam ab97051). The signals were developed by chemiluminescent HRP substrate (Immobilon^TM^ Western, Millipore) and recorded by a digital multispectral imaging system (BioSpectrum, UVP).

## Additional Information

**How to cite this article:** Jiang, Y.- *et al*. Electron tomographic analysis reveals ultrastructural features of mitochondrial cristae architecture which reflect energetic state and aging. *Sci. Rep.* **7**, 45474; doi: 10.1038/srep45474 (2017).

**Publisher's note:** Springer Nature remains neutral with regard to jurisdictional claims in published maps and institutional affiliations.

## Supplementary Material

Supplementary Information

Supplementary Movie S1

Supplementary Movie S2

Supplementary Movie S3
