# Supplementary material for: Electron tomographic analysis reveals ultrastructural features of mitochondrial cristae architecture which reflect energetic state and aging
Source: Sci Rep. 2017 Mar 30;7:45474. doi: 10.1038/srep45474 (PMC5371822; doi:10.1038/srep45474)
Supplement: Supplementary Information [file srep45474-s1.pdf]

Electron tomographic analysis reveals ultrastructural features of mitochondrial cristae architecture which reflect energetic state and aging

*Yi-fan Jiang[1,2], Shao-syuan Lin[1], Jing-min Chen[1], Han-zen Tsai[1], Tao-shih Hsieh[1], and Chi-yu Fu[1]**

[1]*Institute of Cellular and Organismic Biology, Academia Sinica, Taipei, Taiwan*

[2]*Graduate Institute of Molecular and Comparative Pathobiology, School of Veterinary Medicine, National Taiwan University, Taipei, Taiwan*

* Corresponding author

Supplementary information

Figure S1. Serial-section electron tomography of a single mitochondrion

(A) 2D micrographs and (B) 3D tomographic slices of serial sections covering an entire volume of a mitochondrion. (C) The joint serial-section tomograms as viewed from the z-axis. Of note, material losses during tissue sectioning left gaps between jointed tomograms.

Figure S2. Western-blot analysis of R_{WT} and R_{M1L}

Western-blot analysis of total protein expression of R_{WT} and R_{M1L} at week 1, 4, 7.

Figure S3. Global disintegration of mitochondrial cristae in aged WT *Drosophila*

(A) Immunofluorescent staining of mitochondria at week 4, 7, 10 and 12 showing larger mitochondria as flies aged. (B) Thin-section EM micrographs of WT IFM at week 10 and 12 showing mitochondrial cristae swelling, vacuolation and disintegration. Scale bar 20 µm (A), 0.5 µm (B).

Figure S4. Mitochondrial protein content in aged WT *Drosophila*

(A) Immuno-EM analysis of ATP synthase, (B) PDHA1 and (C) SOD2 at week 4 and 12. (D) Western-blot analysis of total protein expression at week 4, 7, 10. Scale bar 0.5 µm.

Movie S1. Serial-section electron tomography and segmentation of IFM mitochondrion showing integrated cristae network in 3D

Movie S2. Serial-section electron tomography and segmentation of IFM mitochondrion showing bidirectional matrix confluency in 3D

Movie S3. Serial-section electron tomography and segmentation of a mitochondrion with swirling membranes showing an onion-like core in 3D

Fig S1

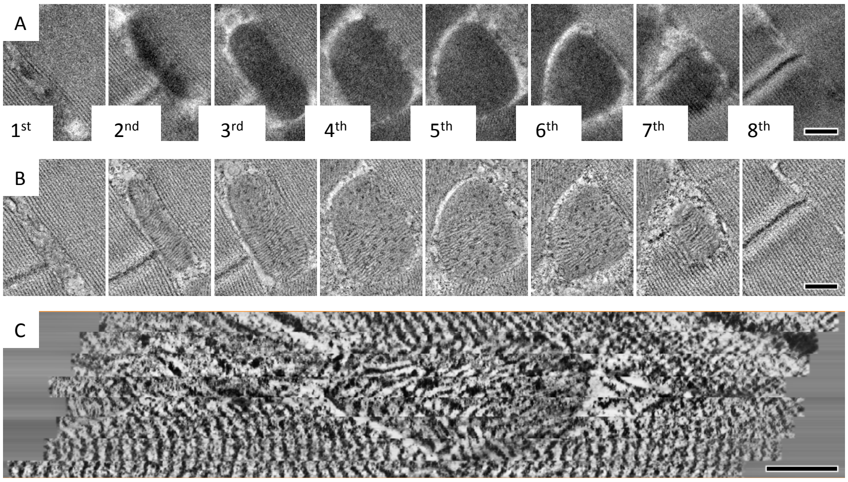

Fig S2

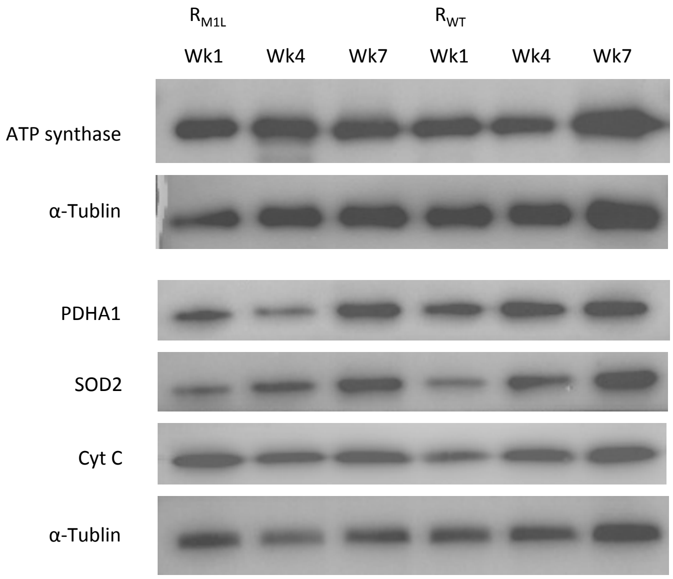
R_{M1L}
R_{WT}
Wk1
Wk4
Wk7
Wk1
Wk4
Wk7
ATP synthase
α-Tublin
PDHA1
SOD2
Cyt C
α-Tublin

Fig S3

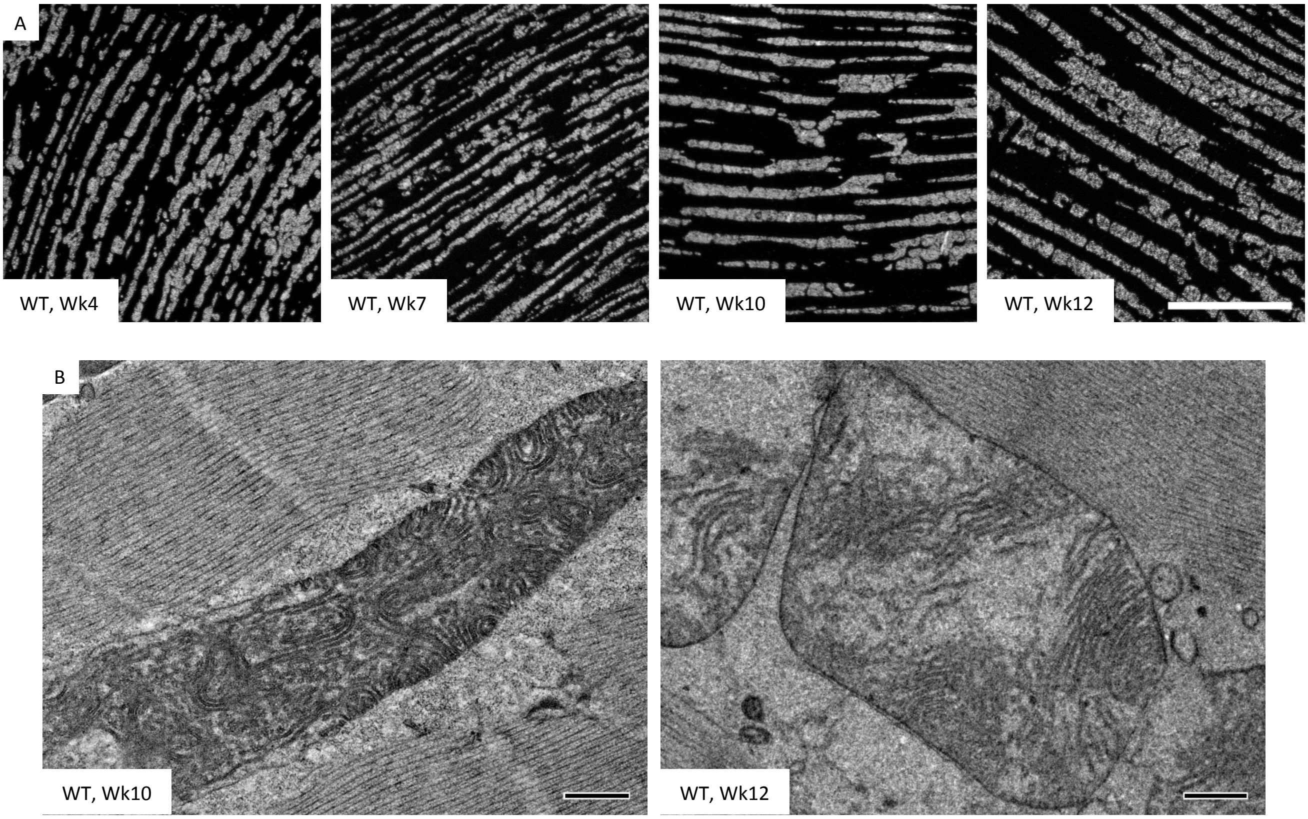

Fig S4

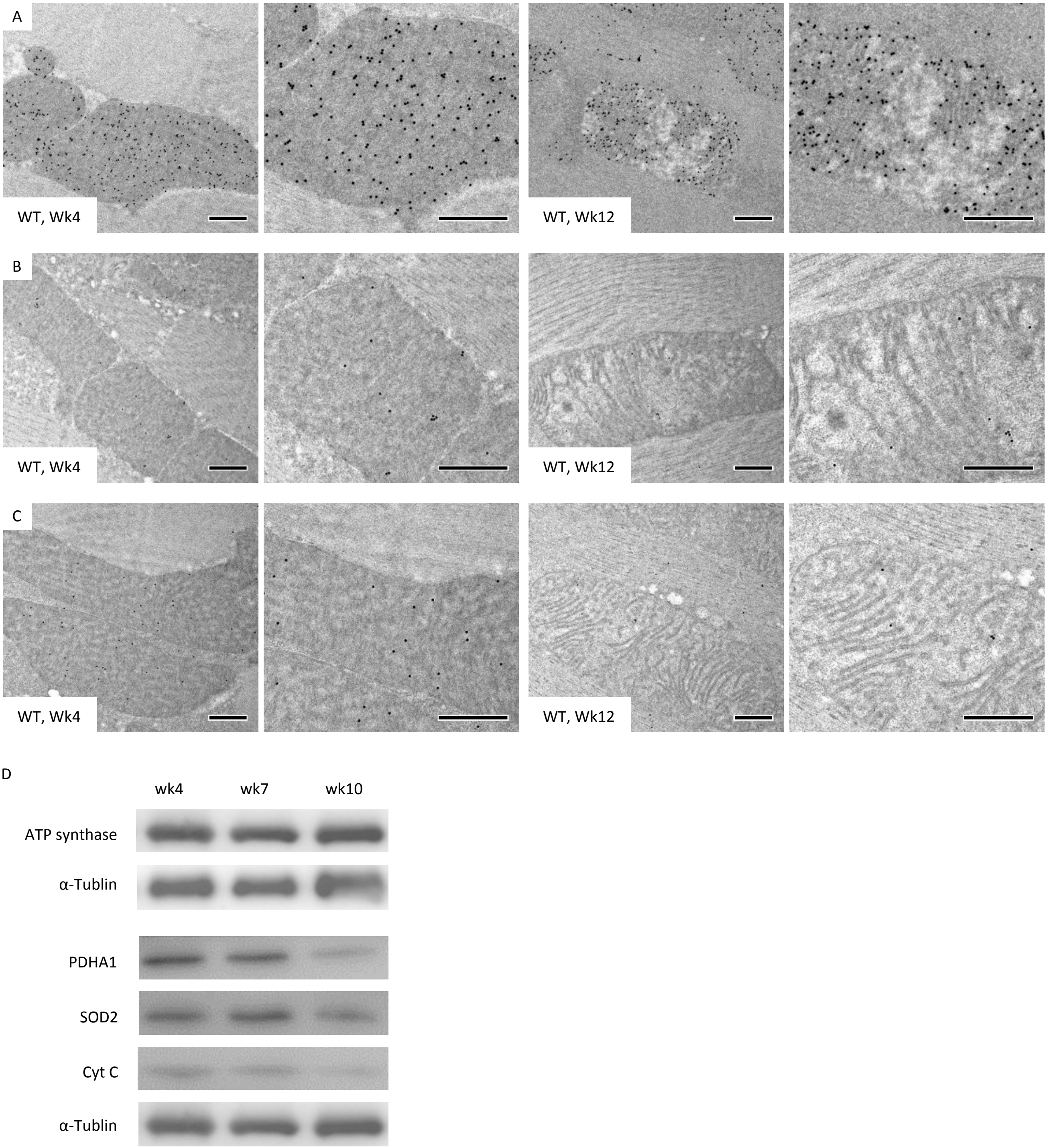
A
WT, Wk4
WT, Wk12
B
WT, Wk4
WT, Wk12
C
WT, Wk4
WT, Wk12
D
wk4
wk7
wk10
ATP synthase
α-Tublin
PDHA1
SOD2
Cyt C
α-Tublin
